# Supplementary material for: Associations of Nutritional, Environmental, and Metabolic Biomarkers with Diabetes-Related Mortality in U.S. Adults: The Third National Health and Nutrition Examination Surveys between 1988–1994 and 2016
Source: Nutrients. 2022 Jun 24;14(13):2629. doi: 10.3390/nu14132629 (PMC9268621; doi:10.3390/nu14132629)
Supplement: Supplementary file 1 [file nutrients-14-02629-s001.zip › nutrients-1773090-supplementary.pdf]

Supplemental Material

Supplemental Table S1. Population characteristics of US adults with diabetes aged over 20 years in NHANES III*

Characteristics	All	Whites	Blacks	Other
Participants (*n*)	2,113	1,392	662	59
Mortality (*n*)	1385	961	394	30
Age (years)	58.5 (0.5)	59.5 (0.6)	55.2 (0.9)	51.6 (1.7)
Female (%)	52.8 (1.8)	50.5 (2.2)	61.9 (2.5)	58.9 (7.6)
Education (%)				
≥ High school	57.8 (2.1)	60.2 (2.5)	49.6 (2.6)	44.0 (6.3)
BMI (kg/m^2)	30.4 (0.2)	30.3 (0.2)	31.3 (0.4)	28.4 (0.5)
BMI (%)				
< 25 kg/m^2	19.6 (1.1)	19.4 (1.3)	15.3(1.5)	42.6 (4.2)
Smoking status (%)				
Nonsmoker	41.2 (1.9)	38.8 (2.3)	47.6 (2.3)	58.9 (5.8)
Past smoker	38.5 (2.0)	41.8 (2.5)	27.0 (1.4)	23.6 (5.7)
Current smoker	20.3 (1.5)	19.3 (1.9)	25.3 (1.8)	17.4 (3.8)
Alcohol intake (%)				
Nondrinker	19.3 (1.4)	16.8 (1.8)	24.3 (2.1)	47.8 (6.7)
Past drinker	49.4 (2.0)	50.4 (2.4)	47.9 (2.0)	35.9 (5.4)
Current drinker	31.3 (2.2)	32.8 (2.6)	27.8 (2.4)	16.6 (2.9)
Physical activity (%)				
Inactive	25.8 (1.2)	23.9 (1.5)	34.1 (2.0)	25.8 (5.3)
Insufficient	43.4 (1.9)	45.5 (2.3)	39.8 (1.8)	19.0 (3.6)
Active	30.8 (1.8)	30.6 (2.1)	26.1 (1.7)	55.2 (6.2)
Diabetes duration (%)				
≥ 10 years	37.5 (2.0)	36.9 (2.4)	41.76 (3.3)	31.4 (10.7)
Comorbidity (%)	48.3 (1.8)	50.1 (2.1)	45.2 (2.8)	26.0 (5.2)
Cancer	12.6 (1.1)	15.1 (1.4)	4.3 (1.0)	0.0 (0.0)
Retinopathy	15.8 (1.4)	14.1 (1.6)	24.5 (2.0)	16.7 (8.8)
Neuropathy	17.5 (1.2)	16.7 (1.5)	21.4 (2.1)	16.0 (4.1)
Chronic kidney disease	17.6 (1.4)	18.0 (1.5)	17.9 (2.1)	9.9 (3.9)
Cardiovascular disease	11.9 (1.2)	12.5 (1.3)	10.2 (1.6)	6.1 (0.4)

*Sample sizes are unweighted. Survey weight-adjusted means (standard error) and percentage (standard error) are presented.

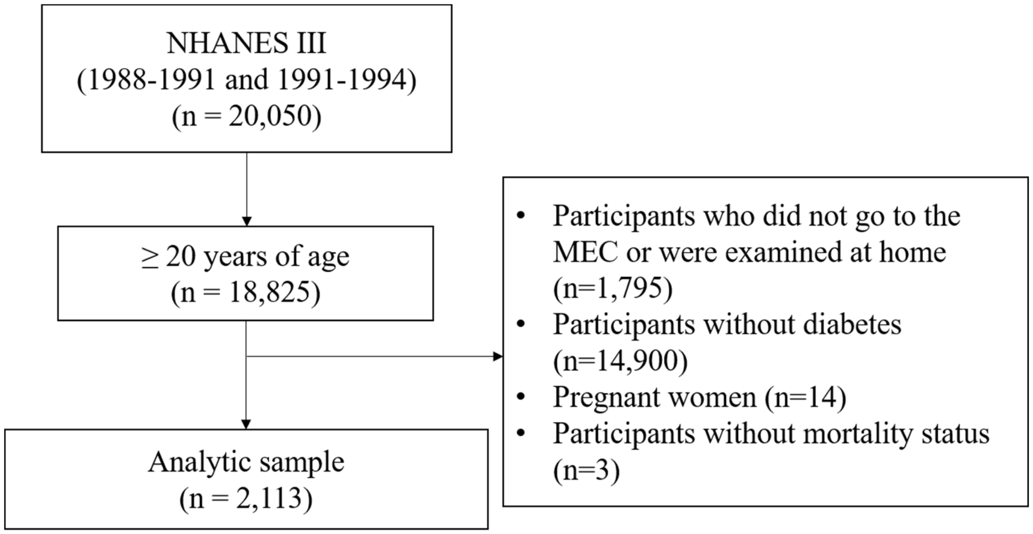

Supplemental Figure S1. Participants selection flow chart. The Third National and Nutrition Examination Survey (NHANES III)

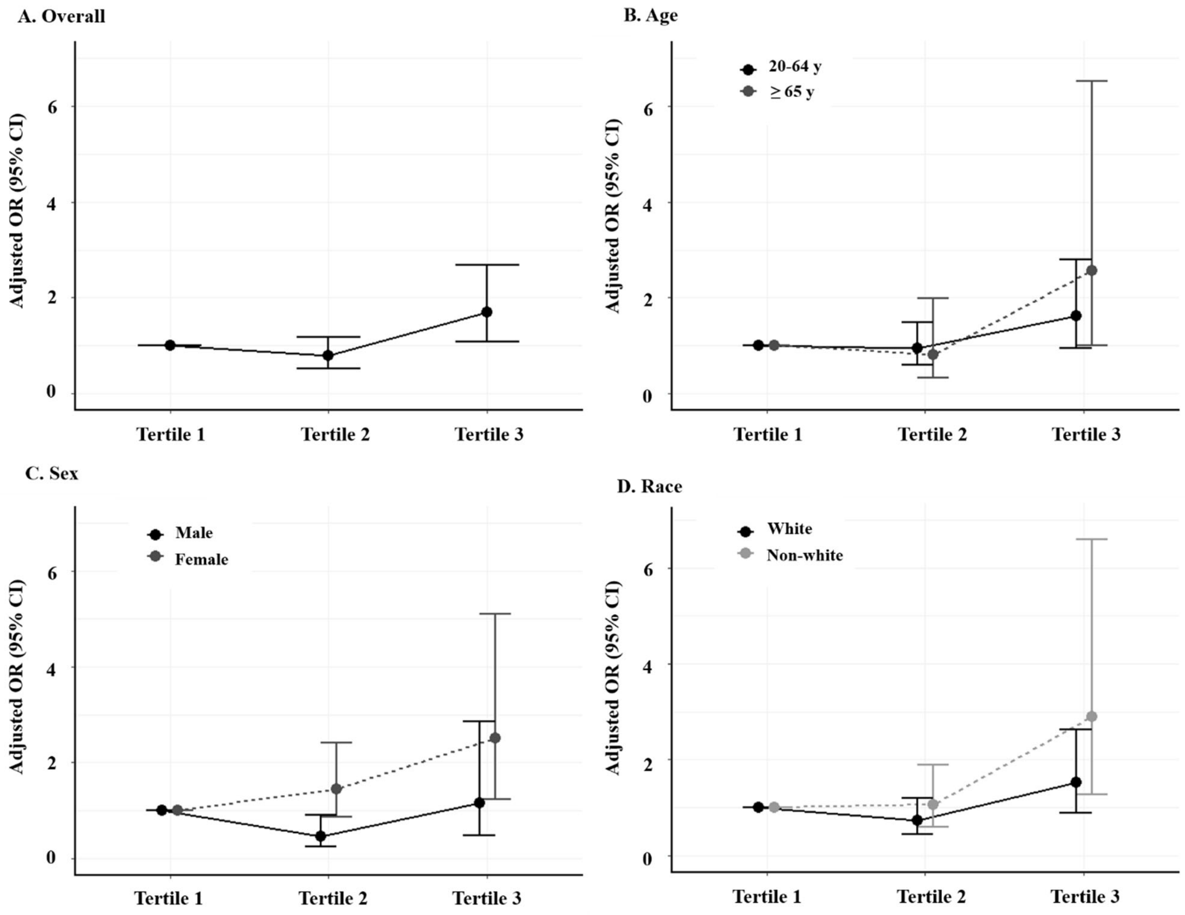

Supplemental Figure S2. Model 2 multivariable-adjusted ORs and 95% CIs of all-cause mortality by tertiles of serum thyroid stimulating hormone biomarker overall (A) and stratified by age, sex, and race (B, C, D).

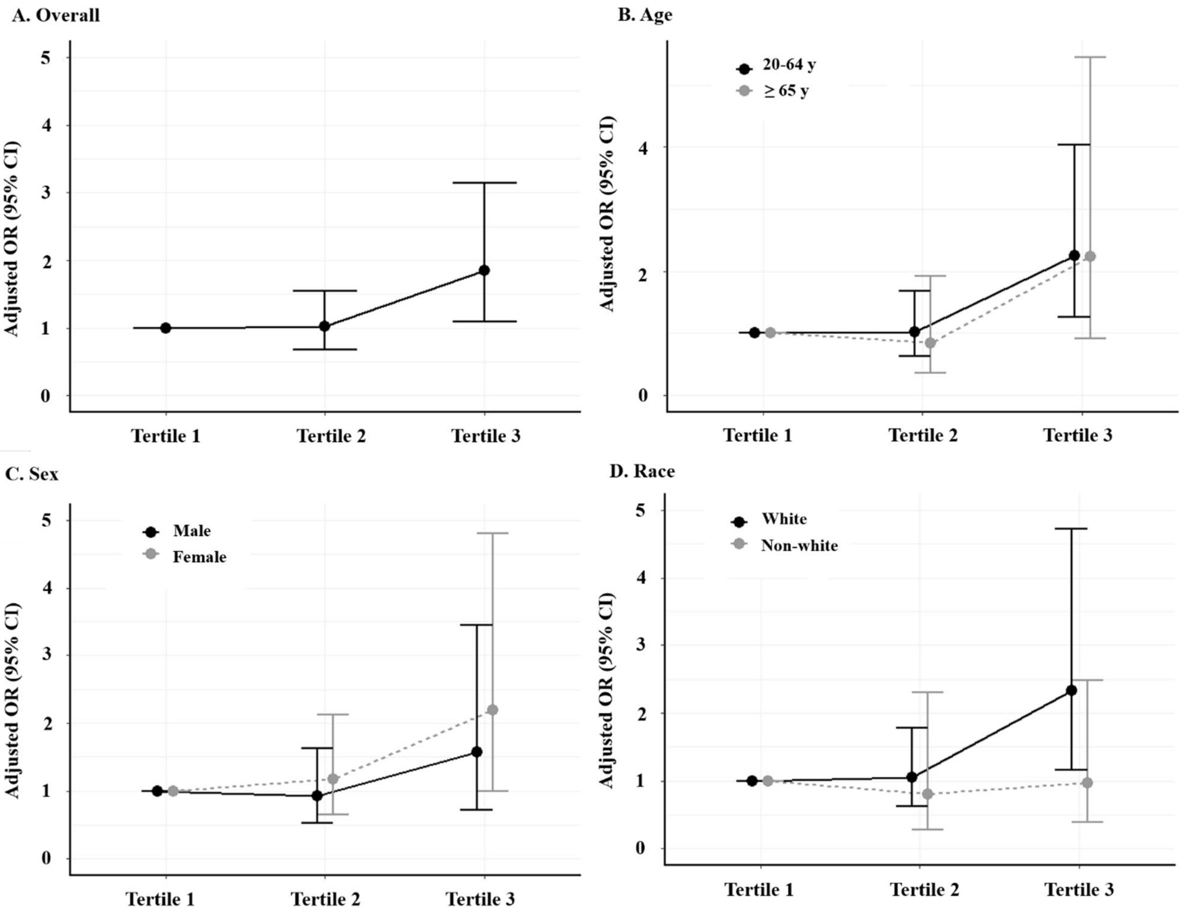

Supplemental Figure S3. Model 2 multivariable-adjusted ORs and 95% CIs of all-cause mortality by tertiles of plasma fibrinogen biomarker overall (A) and stratified by age, sex, and race (B, C, D).

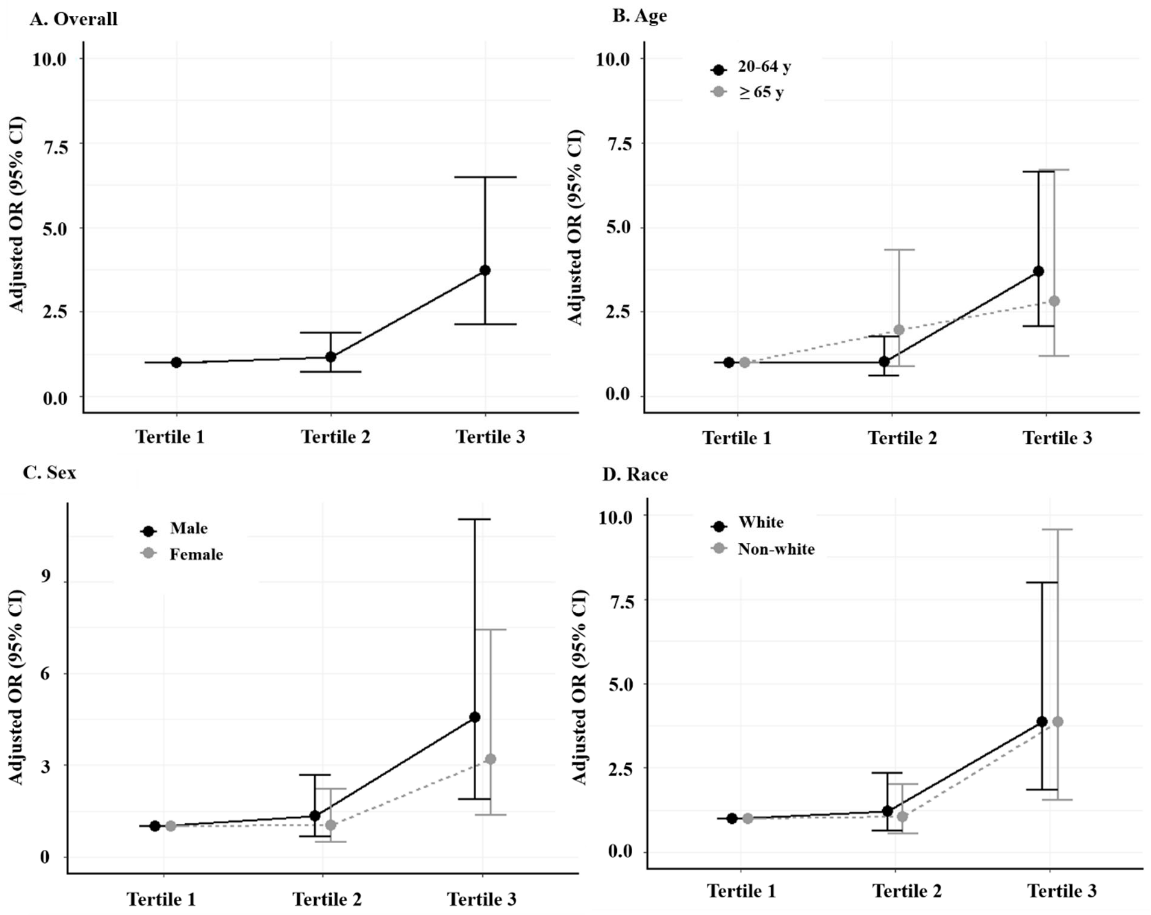

Supplemental Figure S4. Model 2 multivariable-adjusted ORs and 95% CIs of all-cause mortality by tertiles of urine albumin biomarker overall (A) and stratified by age, sex, and race (B, C, D).
